# TNIK drives castration-resistant prostate cancer via phosphorylating EGFR

**DOI:** 10.1016/j.isci.2023.108713

**Published:** 2023-12-12

**Authors:** Jianing Guo, Jiaming Liang, Youzhi Wang, Tao Guo, Yihao Liao, Boqiang Zhong, Shuyue Guo, Qian Cao, Junbo Li, Amilcar Flores-Morales, Yuanjie Niu, Ning Jiang

**Affiliations:** 1 Department of Urology, Tianjin Institute of Urology. The Second Hospital of Tianjin Medical University, Tianjin 300211, China; 2 Department of Pathology, The Second Hospital of Tianjin Medical University, Tianjin 300211, China; 3 Department of Diagnostic and Therapeutic Ultrasonography, Tianjin Medical University Cancer Institute and Hospital, National Clinical Research Center of Cancer, Key Laboratory of Cancer Prevention and Therapy, Tianjin 300211, China; 4 Department of Drug Design and Pharmacology, Københavns Universitet, Faculty of Health and Medical Sciences, University of Copenhagen, 2200 Copenhagen, Denmark

**Keywords:** Biochemistry, Molecular biology, Cancer

## Abstract

The development of castration-resistant prostate cancer (CRPC) is driven by intricate genetic and epigenetic mechanisms. Traf2- and Nck-interacting kinase (TNIK) has been reported as a serine/threonine kinase associated with tumor cell proliferation or unfavorable cancer behavior. The microarray approach revealed a substantial upregulation of TNIK expression levels, enabling us to investigate the functional behaviors of the TNIK gene in CRPC. Specifically, we discovered that AR suppresses TNIK gene transcription in LNCaP and C4-2 cells by forming a complex with H3K27me3. Following the reduction of AR levels induced by androgen deprivation therapy (ADT), TNIK is recruited to activate EGFR signaling through phosphorylation in C4-2 cells, thereby promoting CRPC progression. Our findings unveil a regulatory role of AR as a repressor for TNIK while also highlighting how TNIK activates the EGFR pathway via phosphorylation to drive CRPC progression. Consequently, targeting TNIK may represent an appealing therapeutic strategy for CRPC.

## Highlights

• Inhibition of AR by ADT therapy leads to an increase in TNIK
• TNIK activates transcription of EGFR by acting on its ECD domain
• NCB-0846 can regulate ferroptosis, EMT, and bone metastasis in prostate cancer

## Graphical abstract

**Figure:**
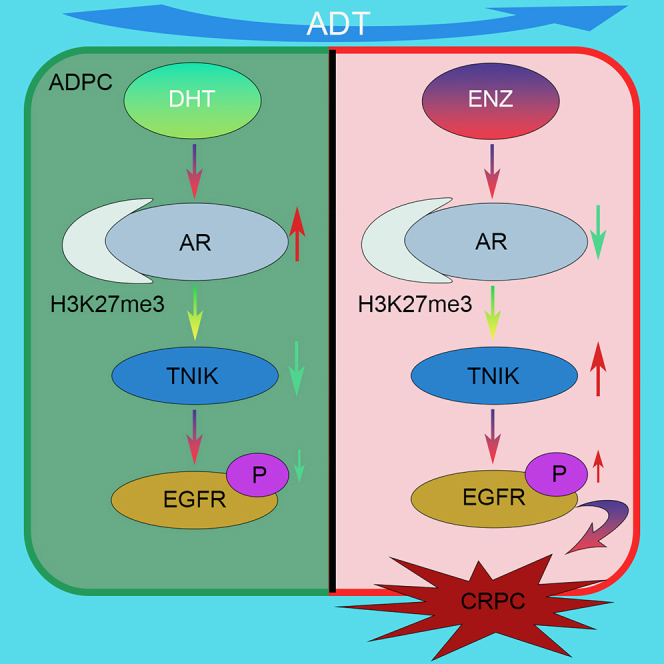

## Introduction

The prevalence of CRPC continues to be the leading cause of mortality among male patients worldwide.^1^^,^^2^ The rapid progression, ease of transfer, and development of late castration resistance of CRPC pose challenges to treatment.^3^^,^^4^ The existence of androgen-repressed genes has been reported in a few studies, with some suggesting their involvement in the progression of CRPC through indirect mechanisms.^5^^,^^6^^,^^7^^,^^8^^,^^9^ The identification of targets is imperative for the advancement of CRPC development.^10^

Traf2- and Nck-interacting kinase was initially identified as germinal center kinases (GCKs) and plays a crucial role in regulating various fundamental cellular processes through phosphorylation of its downstream substrates.^11^^,^^12^ The proto-oncoprotein TNIK exhibits overexpression in various malignancies, including prostate cancer (PCa), multiple myeloma, pancreatic cancer, hepatocellular carcinoma, and gastric cancer.^13^^,^^14^^,^^15^^,^^16^^,^^17^^,^^18^ For example, TNIK protein phosphorylates AKT and promotes the proliferation of gastric cancer cells.^19^ Furthermore, TNIK-deficient mice showed reduced expression of Myc and Cd44.^20^ TNIK was also shown to be involved into the NF-κB and SMAD signaling pathways.^11^^,^^17^

Emerging evidence has demonstrated the crucial involvement of epidermal growth factor receptor (EGFR) signaling pathways in prostate regulatory mechanisms during prostate tumorigenesis. Activation of the EGFR signaling pathway plays a pivotal role in promoting cancer cell survival under androgen-depleted conditions. Upregulation of EGFR signaling may be a mechanism by which PC cells escape castration-induced cell death.^21^ The association between TNIK and EGFR, however, remains unestablished in existing literature.

In this study, we employed GeneArray analysis to elucidate the mechanism underlying the development of CRPC. Our findings revealed TNIK as a potential driver gene for CRPC that interacts with EGFR. Notably, our evidence demonstrated that AR directly repressed TNIK transcription through the AR-H3K27me3 complex. Upon si-AR or MDV3100 treatment, TNIK gene expression was activated, leading to its binding to the extracellular domain (ECD) of EGFR and subsequent promotion of phosphorylation. Previous studies have implicated the ECD domain of EGFR in cellular drug resistance, suggesting that the interaction between TNIK and ECD may play a crucial role in CRPC emergence.^22^ The activated EGFR is mainly located in the nucleus and regulates the transcription of target genes, thereby promoting the progression of CRPC. Pharmacological inhibitors of TNIK (NCB-0846) inhibited the growth of CRPC cell xenografts. Furthermore, we observed that silencing TNIK also enhances EGFR-mediated, erastin-induced ferroptosis in CRPC cells. This kinase represents a promising candidate for targeted drug therapy in CRPC and provides valuable insights for potential combination strategies involving ferroptosis-based therapies.

## Results

### TNIK was upregulated in castration-resistant prostate cancer

**Figure 1:**
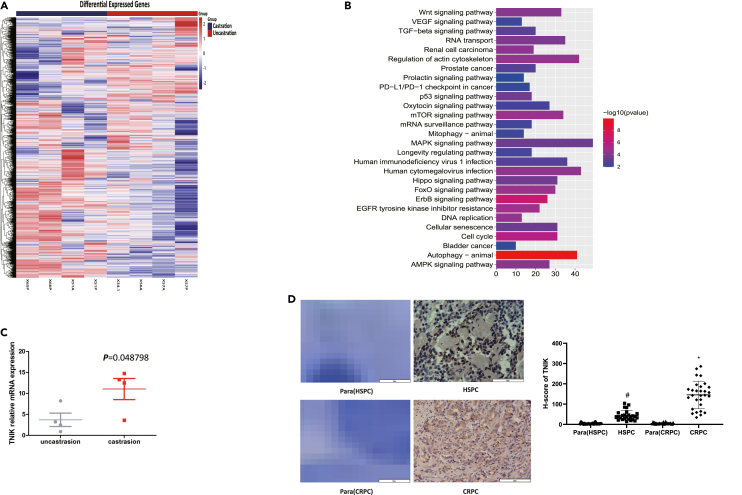
TNIK was upregulated in castration-resistant prostate cancer (A) Heatmap of the genome-wide transcript profile between (CR-LNCaP) castration tumors and (HS-LNCaP) uncastration tumors. (B) GO analysis of transcript profile. (C) qPCR detects mRNA expression of TNIK in (CR-LNCaP) castration tumors and (HS-LNCaP) uncastration tumors (p < 0.05). Data are represented as mean ± SD. (D) Clinical specimens of benign prostate hyperplasia (BPH), hormone native, and CRPC were analyzed by IHC for TNIK expression. Scale bars: 200 μm. H-score of TNIK immunoreactivity in HSPC, CRPC, and their paracancerous tissues. Data were presented as median (interquartile range). Data are represented as mean ± SD. The hash symbol indicates significantly different from Para (HSPC) group (p < 0.05); an asterisk indicates significantly different from Para (CRPC) group (p < 0.05).

Previously, LNCaP cells were cultured under androgen deprivation conditions using 10% Certified FBS Charcoal Stripped and RPMI Medium 1640 culture medium. After about four months of culture and multiple passages, the CR-LNCaP cell line was obtained. Subsequently, a model of castration-resistant LNCaP tumors (CR-LNCaP) and androgen-sensitive tumors (HS-LNCaP) was established with LNCaP xenografts.^8^ In order to identify unrecognized molecular mechanisms of CRPC between intact and castrated mice, we quantified changes in mRNA levels of human genes in HS/CR-LNCAP tumors. We found that 1,884 genes were upregulated and 588 genes were repressed in CR-LNCaP compared with HS-LNCaP tumors (log_2_FC > 1.5, p < 0.05) (Figure 1A; Table S1). GO analysis revealed a significant enrichment of upregulated differentially expressed genes involved in mRNA processing and nuclear transport signaling pathway (Figure 1B). Among these genes, we focused on TNIK serine/threonine kinase due to its upregulated expression in CRPC associated with the EGFR signaling pathway. To validate the microarray results, we performed qPCR analysis on xenograft tumor samples from CR-LNCaP (4 castrations) and HS-LNCaP (4 uncastrations), confirming higher mRNA expression of TNIK in CR-LNCaP compared with HS-LNCaP (Figure 1C). Furthermore, we investigated the potential role of TNIK by analyzing prostatectomy samples obtained from patients with hormone-sensitive prostate cancer (HSPC) (26 cases), CRPC (29 cases), and their respective paracancerous tissues using immunohistochemistry staining for TNIK expression patterns (Figure 1D). The results showed that both cytoplasmic and nuclear compartments exhibited circumscribed expression of TNIK within the epithelial cells, with a progressively significant increase observed in CRPC samples. These findings are consistent with the data obtained from microarray analysis shown in Figures 1C and 1D, indicating increased mRNA and protein expression of TNIK in CRPC cells.

### Knockdown TNIK inhibited CRPC cell proliferation

**Figure 2:**
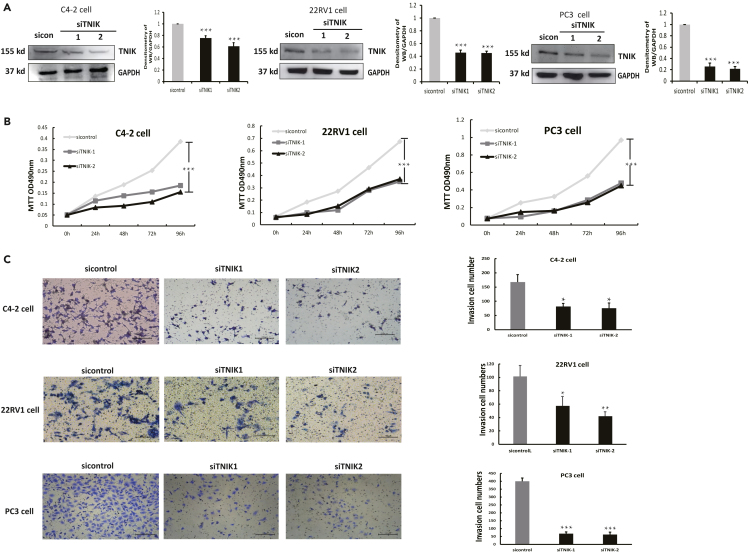
Knockdown TNIK inhibited CRPC cell proliferation (A) C4-2, 22RV1, and PC3 cells were transfected with TNIK siRNA and lysed 48 h posttransfection. Whole-cell extracts were analyzed by western blotting with the indicated antibodies. Data are shown as mean ± SD. ∗∗∗p < 0.005. (B) C4-2, 22RV1, and PC3 cells were transfected with siControl or siTNIK, MTT detected cell viability. Data are shown as mean ± SD. ∗∗∗p < 0.005. (C) C4-2, 22RV1, and PC3 cells were transfected with siControl or siTNIK; transwell detected cell invasion. Scale bars: 100 μm. Significance was determined by a two-tailed t test: Data are shown as mean ± SD. ∗p < 0.05, ∗∗p < 0.01, ∗∗∗p < 0.005.

To investigate the impact of TNIK on cell proliferation in CRPC, siRNA targeting TNIK was transfected into CRPC cells (C4-2, 22RV1, and PC3). Efficient knockdowns of TNIK were successfully achieved in these cell lines (Figure 2A). Subsequently, we observed a clear inhibition of proliferation in C4-2, 22RV1, and PC3 cells upon downregulation of TNIK (Figure 2B). Furthermore, the reduction of TNIK expression significantly attenuated the invasive C4-2, 22RV1, and PC3 cells as demonstrated by Matrigel assays (Figure 2C). These findings strongly support that TNIK plays a crucial role in promoting cell proliferation within CRPC. In summary, our results highlight the independent functions exerted by TNIK in androgen-independent PC cells while emphasizing its pivotal contribution to the growth and metastasis of castration-resistant tumors.

### Androgen-AR signaling suppressed TNIK gene expression

**Figure 3:**
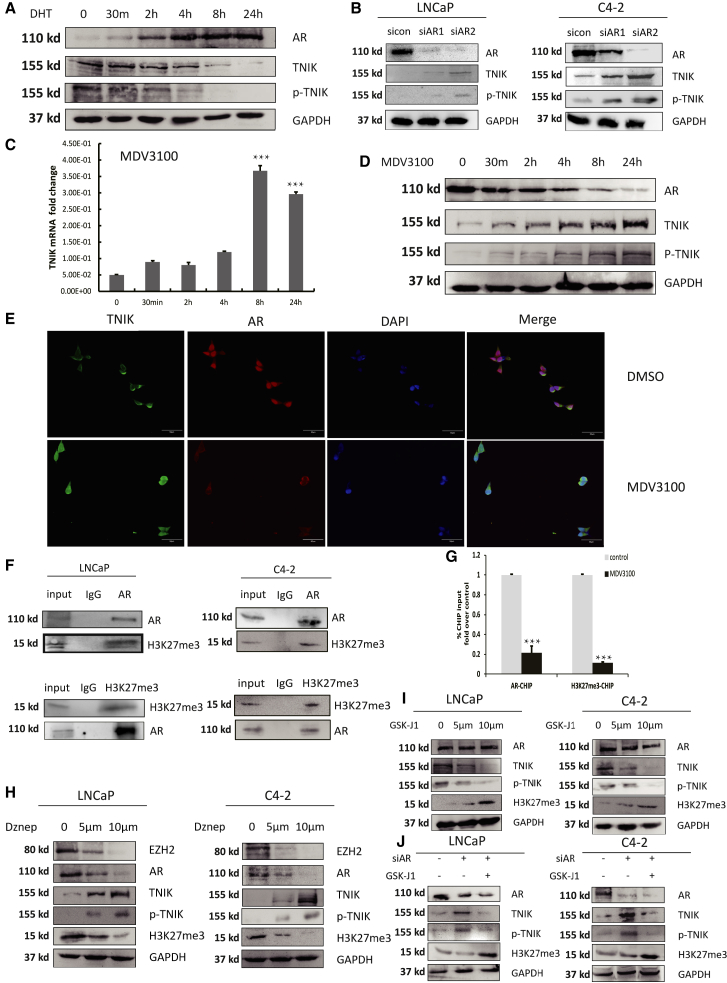
The essential role of the AR and H3K27me3 complex in the regulation of TNIK expression (A) LNCaP cells were cultured in steroid-depleted medium for 24 h and subsequently treated with DHT (10 nM) for the indicated time. Protein levels were analyzed by western blot. (B) The levels of TNIK protein were measured by western blot in LNCaP and C4-2 cells upon AR knockdown. (C) qPCR analysis mRNA expression of TNIK after LNCaP cells were treated with MDV3100 for 24 h. Data are shown as mean ± SD. ∗∗∗p < 0.005. (D) The levels of TNIK protein were measured in LNCaP cells treated for 24 h with the AR antagonist MDV3100 (enzalutamide, 100 nM). (E) Coimmunofluorescence (co-IF) analysis of TNIK and AR proteins in LNCaP treated with MDV3100. Scale bars: 50 μm. (F) Immunoprecipitation of AR or H3K27me3 in LNCaP and C4-2 cells followed by immunoblot analysis of H3K27me3 or AR. IgG represents a control antibody used for IPs. (G) ChIP–PCR analysis of H3K27me3 and AR binding to the TNIK gene promoter in LNCaP cells treated with MDV3100. ChIP–PCR primer in Table S3. Data are shown as mean ± SD. ∗∗∗p < 0.005. (H and I) LNCaP and C4-2 cells were treated with DZNep (0, 5, 10 μm) and GSKJ1 (0, 5, 10 μm) for 8 h, WB analysis of TNIK protein expression. (J) Western blot showed that treatment of the indicated LNCaP or C4-2 cells with GSK-J1 could reverse the effects of AR knockdown on TNIK.

The upregulated expression of TNIK in CR-LNCaP compared with HS-LNCaP tumors suggests a potential regulation of TNIK by androgens. Therefore, we investigated the impact of dihydrotestosterone (DHT) treatment (10 nM) on TNIK levels. A time-course study of DHT incubation in LNCaP cells revealed that androgen treatment suppressed the expression and phosphorylation of TNIK (Figure 3A). We observed increased TNIK protein expression and phosphorylation levels upon AR knockdown in PCa cells, whereas treatment of LNCaP cells with the AR antagonist MDV3100 also significantly upregulated TNIK mRNA and protein levels, as well as phosphorylation levels of TNIK (Figures 3B–3D). Consistent with the upregulation of TNIK detected in CR-LNCaP, we also observed that inhibition of androgen exposure led to increased nuclear abundance of TNIK through immunofluorescence imaging (Figure 3E). In our previous study, we reported a cooperative role between EZH2 and AR for YAP1 transcriptional repression.^23^ The sole identified methyltransferase with activity toward H3K27, EZH2, is solely responsible for all methylation of H3K27.^24^ Therefore, we performed co-immunoprecipitation (Co-IP) experiments to verify whether there is an interaction between AR and H3K27me3 in PCa cells. As depicted in Figure 3F, AR formed a stable complex with H3K27me3 in PCa cells. The specificity of these protein interactions was confirmed, as no visible interaction was observed in the IgG control. Furthermore, chromatin immunoprecipitation experiments demonstrated that both AR and H3K27me3 were recruited to the TNIK gene promoter; however, treatment with MDV3100 abolished the ability of AR to form a complex (Figure 3G). Treatment of PCa cells with DZNeP (EZH2 inhibitor) resulted in an increase in TNIK protein expression as well as phosphorylation levels, while interestingly downregulating AR levels as well (Figure 3H). In order to validate the role of H3K27me3 in transcriptional repression by AR, we utilized GSK-J1 (H3K27 demethylase inhibitor) to elevate the level of H3K27me3. We observed a decrease in TNIK expression when H3K27me3 levels increased, suggesting the inhibitory effect of H3K27me3 on TNIK, whereas the non-significant change in AR expression levels confirmed that EZH2-mediated regulation of AR levels is independent of its methylation function, consistent with previous studies (Figure 3I).^25^ In addition, the elevation of H3K27me3 effectively counteracted the promoting effect of AR downregulation on TNIK expression (Figure 3J). Overall, these experiments demonstrated that the AR and H3K27me3 complex mediated the androgen-driven epigenetic repression of TNIK.

### TNIK interacts directly with EGFR

**Figure 4:**
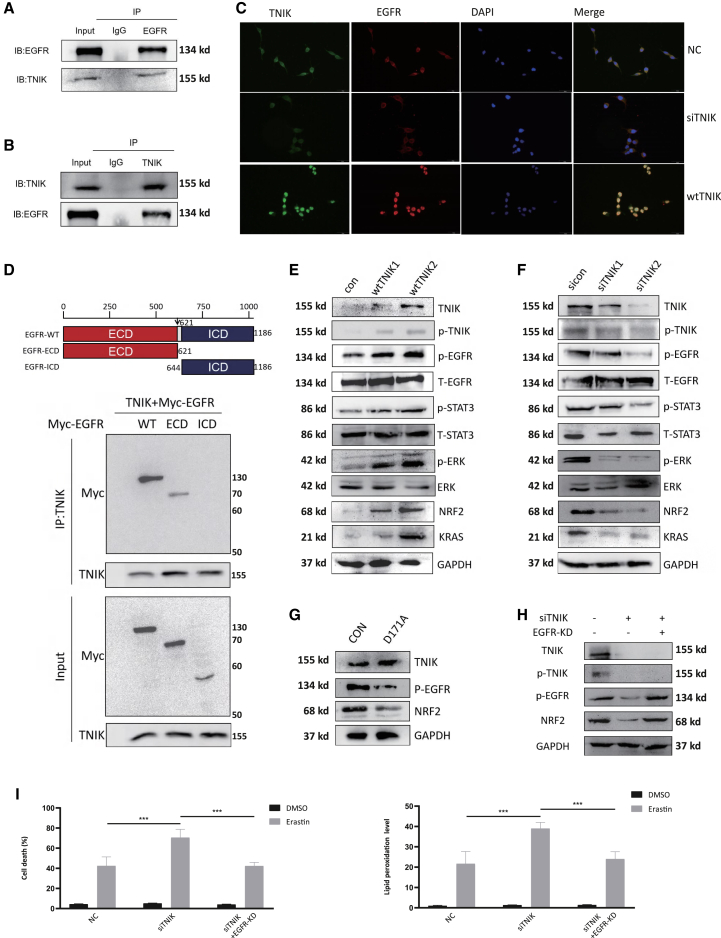
TNIK binds to the ECD domain to phosphorylate EGFR and regulate ferroptosis resistance (A) Immunoprecipitation of TNIK in C4-2 cells followed by immunoblot analysis of EGFR or TNIK. (B) Immunoprecipitation of EGFR in C4-2 cells followed by immunoblot analysis of EGFR or TNIK. (C) Co-immunofluorescence (co-IF) analysis of TNIK and EGFR proteins in C4-2 cells after transfected TNIK wild-type vector. Scale bars: 50 μm. (D) Schematic diagram of the Myc-EGFR constructs. C4-2 cells were transfected with the indicated plasmids for 48 h. The cell lysates were immunoprecipitated with anti-TNIK immunomagnetic beads and immunoblotted with the indicated antibodies. (E) The levels of EGFR/*p*-EGFR and EGFR-related protein were measured in C4-2 cells after transfected TNIK wild-type vector. (F) The levels of EGFR/*p*-EGFR and EGFR-related protein were measured in C4-2 cells after transfected siTNIK. (G) Expression of a TNIK kinase mutant (D171A) reduces phosphorylation of EGFR. (H) The levels of *p*-EGFR and NRF2 protein were measured in C4-2 cells after knocking down the ECD domain. (I) Levels of cell death in C4-2 cells treated with 4 μm erastin (up). Levels of lipid peroxidation in C4-2 cells following the same treatment (down). Data are shown as mean ± SD. ∗∗∗p < 0.005.

Gene set enrichment analysis (GSEA) was conducted to investigate the involvement of activating EGFR pathways in LNCaP-CR vs. LNCaP-HS, where the differential expression profile of genes was analyzed (Table S2). Although the role of TNIK in certain cancers has been extensively documented, its role in CRPC remains less understood. To validate the regulatory function of TNIK on EGFR expression, we utilized C4-2 cell line with an active EGFR pathway. The interaction between TNIK and EGFR was examined in C4-2 cells by immunoprecipitating TNIK from lysates and probing for interaction with EGFR through western blotting (Figures 4A and 4B). As expected, direct binding between TNIK and EGFR was observed. Furthermore, using immunofluorescence imaging, we found that overexpression of TNIK led to increased abundance of nuclear-localized EGFR, whereas decreased levels of TNIK resulted in reduced nuclear import of EGFR (Figure 4C). In order to determine the binding domain between TNIK and EGFR more precisely, we generated several plasmids expressing truncated versions of Myc-tagged-EGFR (Myc-EGFR). Immunoprecipitation experiments were performed in C4-2 cells coexpressing these Myc-tag mutants along with TNIK. Results indicated that when the ECD domain was deleted from these mutants, they were not detected in the immunoprecipitates pulled down by anti-Myc immunomagnetic beads (Figure 4D). Next, we investigated the impact of TNIK overexpression or siRNA-mediated knockdown on EGFR phosphorylation and its downstream target genes. Our findings revealed that TNIK overexpression activated EGFR phosphorylation and its associated signaling pathways, including the ferroptosis-related gene NRF2. Conversely, knockdown of TNIK had the opposite effect (Figures 4E and 4F). Mutants with attenuated catalytic activity at S171 exhibited reduced EGFR phosphorylation and NRF2 upregulation compared with mutant controls (Figure 4G). To further validate these results, we suppressed the activity of the ECD domain in EGFR using a plasmid. We observed that deletion of the ECD domain reversed the inhibitory effect of TNIK knockdown on EGFR phosphorylation and the changes in EGFR-associated NRF2 levels (Figure 4H). This further illustrates the importance of the ECD domain for TNIK to bind and function with EGFR. Notably, knockdown of TNIK decreased C4-2 cell resistance to erastin (a ferroptosis inducer). Similar to previous results, the ferroptosis sensitivity caused by TNIK knockdown was reversed when the ECD domain was deleted. (Figure 4I). In conclusion, our study highlights the essential role of TNIK in optimal EGFR phosphorylation and transcriptional activation while emphasizing its regulatory effects on ferroptosis as a potential avenue for combination ferroptosis therapies.

### TNIK inhibitor inhibited proliferation and invasion of CRPC cell

**Figure 5:**
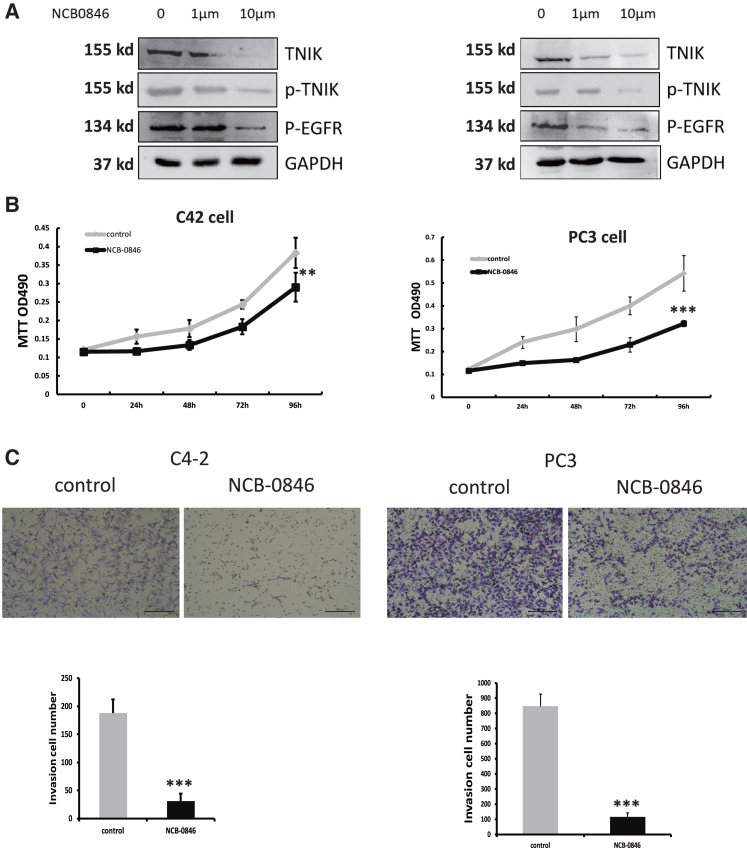
TNIK inhibitor inhibited proliferation and invasion of CRPC cell (A) C4-2 and PC3 cells were treated with NCB-0846 24 h. Whole-cell extracts were analyzed by western blotting with the indicated antibodies. (B) C4-2 and PC3 Cells were seeded into 96-well plates and treated with NCB-0846 (10 μm); MTT detected cell viability. Data are shown as mean ± SD. ∗∗p < 0.01, ∗∗∗p < 0.005. (C) C4-2 and PC3 cells were treated with NCB-0846 (10 μm); transwell detected cell invasion ability. Scale bars: 100 μm. Data are shown as mean ± SD. ∗∗∗p < 0.005.

The efficacy of the small molecule TNIK inhibitor NCB-0846 on tumor cells has been confirmed.^13^^,^^26^ To test the efficacy of NCB-0846 during prostate cancer treatment and in CRPC, we initially assessed the efficacy of NCB-0846 in suppressing TNIK protein expression in C4-2 and PC3 cells. Treatment with 1 μm or 10 μm of NCB-0846 significantly reduced the levels of TNIK protein and phosphorylation after 24 h (Figure 5A). Furthermore, western blot analysis revealed a concurrent decrease in *p*-EGFR activity upon TNIK downregulation following inhibitor treatment in both C4-2 and PC3 cells. Notably, C4-2 and PC3 cells exhibited sensitivity to NCB-0846 treatment at a concentration of 10 μm (Figure 5B). Additionally, NCB-0846 treatment effectively inhibited cell invasion in both C4-2 and PC3 cells at a concentration of 10 μm (Figure 5C). Collectively, these findings highlight the potential therapeutic targeting of TNIK as an innovative approach for treating CRPC by modulating the EGFR signaling pathway.

### Targeting TNIK suppressed CRPC tumor progression *in vivo*

**Figure 6:**
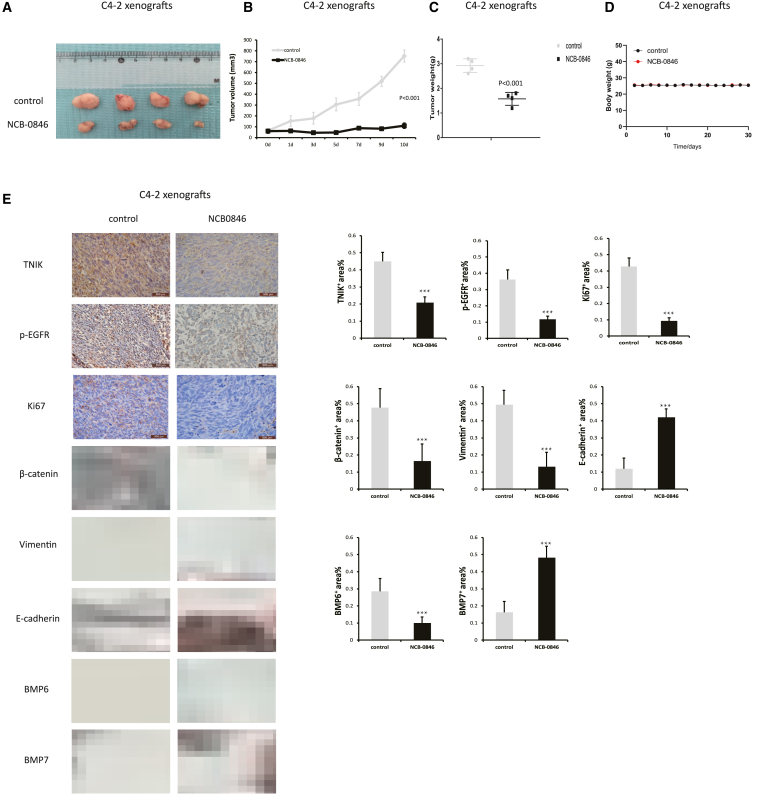
Targeting TNIK suppresses CRPC tumor progression *in vivo* (A) C4-2 cells were implanted subcutaneously in male BALB/c mice. When tumors became palpable, mice administered daily by oral gavage either with vehicle (10% DMSO in PBS) or NCB0846 (80 mg/kg of body weight) for 10 days (n = 4 mice for each treatment). Tumor volumes were measured with calipers. (B) Tumor size of xenografts of the above represented the growth of tumor over 10 days (n = 4) in athymic nude mice (p < 0.001). Data are shown as mean ± SD. (C) Tumor weight of the control mice tumors and NCB-0846-treated mice tumors (p < 0.001). Data are shown as mean ± SD. (D) Body weight of nude mice after implantation of control or C4-2 xenografts and treatment with vehicle or NCB-0846 for 4 weeks. (E) Quantitation of Ki-67, TNIK, *p*-EGFR, β-catenin, vimentin, E-cadherin, BMP6, and BMP7 expressions in C4-2 xenograft tumors from each group; specimens were got at 10 days posttreatment. Scale bars: 500 μm. The IHC was scored according to number of cells expressing the indicated proteins, and statistical analysis was performed (non-parametric Kruskal-Wallis test) in order to determine significance. Data are shown as mean ± SD. ∗∗∗p < 0.005.

To investigate the impact of the TNIK inhibitor on the growth of CRPC xenograft tumors, 2 × 10^6^ C4-2 and PC3 cells were subcutaneously implanted in BALB/c mice. Once the tumors reached approximately 100 mm^3^ in size, the mice were randomly assigned to receive either vehicle (10% DMSO in PBS) or NCB0846 (80 mg/kg of body weight) daily via oral gavage for a duration of 10 days (n = 4 mice per treatment group). While robust subcutaneous CRPC tumors formed in the DMSO-treated mice, tumor growth was noticeably smaller in the NCB-0846-treated group (Figures 6A and S1A). A significant inhibition of tumor growth was observed in the NCB-0846-treated mice compared with those treated with DMSO alone (Figures 6B, 6C, S1B, and S1C). Importantly, administration of NCB-0846 did not result in any apparent toxicity, as it had no effect on body weight changes (Figures 6D and S1D). Furthermore, we found that tumors treated with NCB-0846 exhibited reduced TNIK expression levels and decreased Ki67 and *p*-EGFR expression (Figures 6E and S1E). Because previous articles have demonstrated that EGFR is critical for the regulation of EMT in PCa, we speculate that TNIK can also regulate the EMT process through EGFR.^27^ Therefore, we tested three EMT markers (β-catenin, vimentin, and E-cadherin) and found that inhibiting TINK seemed to block EMT in PCa. More importantly, in order to further explore the therapeutic potential of TNIK-targeted therapy in PCa, we detected two more bone metastasis markers (BMP6, BMP7) in PCa. Surprisingly, NCB-0846 also had inhibitory effects on PCa bone metastasis. These findings underscored the potential therapeutic value of targeting TNIK signaling to enhance sensitivity toward CRPC therapy and inhibit its progression and metastasis.

## Discussion

In the present study, we have elucidated crucial components of the interplay between the AR and EGFR signaling pathways via TNIK. By selectively inhibiting TNIK to impede EGFR phosphorylation, we effectively suppressed the proliferation of CRPC.

The previous study reported that TNIK was frequently upregulated in high-grade ovarian cancer tumors and serous hepatocellular carcinoma.^18^ Moreover, TNIK hyperactivity contributed to human lung adenocarcinoma cell metastasis.^17^ In this study, we initially identified TNIK as a potential biomarker of CRPC by analyzing gene array files from mouse models. We observed elevated expression levels of TNIK not only in CR-LNCaP tumors in mice but also in CRPC patients compared with those with localized PCa and benign prostatic hyperplasia (BPH) (Figure 1D). These findings suggest a correlation between TNIK and aggressive behavior in cancer.

The principal findings of our study revealed that AR forms a repressive complex with H3K27me3 at the TNIK promoter, leading to the suppression of TNIK transcription (Figure 3). Consequently, ADT induces TNIK mRNA expression, which in turn regulates EGFR phosphorylation to activate the EGFR signaling pathway and contribute to CRPC growth. The transcriptional activity of AR is regulated by interacting coactivators that positively modulate receptor function. Conversely, AR inhibits target gene expression through "corepressors" such as Alien, SMRT, and NCoR.^28^^,^^29^^,^^30^ A few reports have studied AR inhibition of transcription. Some hinted to indirect mechanisms—DNA methylation or protein phosphorylation^23^^,^^31^—whereas others hinted to direct mechanisms in involving the epigenetic silencing complex.^24^^,^^32^ Using TNIK as a model, we discovered that AR can also suppress gene expression through hormone-induced recruitment of H3K27me3 to the AR/H3K27me3 complex, thereby directly inhibiting transcription. Consequently, ADT restores TNIK expression by disrupting the association between AR and H3K27me3 at the TNIK promoter.

EGFR signaling pathway also plays a crucial role in CRPC. Substantial evidence has accumulated, indicating that the expression of EGFR is associated with an increased risk of high-grade, advanced disease, as well as prostate-specific antigen recurrence.^32^ Castration of mature animals can increase the expression of EGFR protein in the prostate in a time-dependent manner.^33^ Our findings also suggest that castration resistance may be induced by reciprocal interaction between TNIK and the EGFR signaling pathway, with TNIK playing a major role in promoting nuclear translocation and activation of EGFR (Figure 4). Phosphorylation of EGFR is crucial for its intracellular distribution and transcriptional activity. ECD domain can recognize and bind to specific ligands, thereby facilitating the activation of EGFR. In this study, we discovered that TNIK can interact with the ECD domain on EGFR to induce phosphorylation and enhance nuclear translocation of EGFR. When the ECD domain is deleted, TNIK loses its regulatory effect on EGFR and EGFR-associated ferroptosis sensitivity. It has been reported that inhibition of TNIK reduces cell viability in ERG-positive PCa cells DU145 and 22RV1.^24^ Our current data further demonstrate that TNIK has the ability to phosphorylate EGFR and promote transcriptional activation of its target genes. The use of TNIK inhibitors has shown promising inhibitory effects in CRPC cells and tumors (Figures 5 and 6).

Interestingly, TNIK also plays a regulatory role in ferroptosis. Ferroptosis is primarily caused by an imbalance between the generation and degradation of intracellular lipid reactive oxygen species (ROS), which is closely associated with cellular metabolic activity. In comparison to normal cells, tumor cells exhibit heightened metabolic rates and consequently face an increased susceptibility to ferroptosis. Previous studies have demonstrated that TNIK regulates glucose and lipid homeostasis in both Drosophila and mice.^34^ The knockout of TNIK has been shown to enhance glucose and lipid metabolism; however, there is currently no research linking TNIK to ferroptosis. Given its role as a crucial regulatory factor in CRPC, the upregulation of TNIK expression may serve as a cellular self-protective mechanism against ferroptosis. Similarly, AR also plays a significant protective role in PCa cells by inhibiting lipid peroxidation and preventing ferroptosis.^35^ When AR is present, in order to sustain the normal metabolic activities necessary for cell growth, AR exerts an inhibitory influence on TNIK. During treatment, the reduction in AR levels triggers the activation of TNIK as a secondary defense mechanism against ferroptosis while concurrently promoting CRPC progression. The collaborative action of AR and TNIK ensures cellular metabolism homeostasis in PCa.

NCB-0846, as a TNIK inhibitor, has shown potential therapeutic effects in a variety of tumors. As a regulatory component of the transcription complex composed of β-catenin and T cell factor 4 (TCF4), TNIK can regulate the Wnt signaling pathway related to stem cell activity. In colorectal cancer, where the Wnt signaling pathway is the most prominent, NCB-0846 has been shown to bind to TNIK in an inactive structure, thereby inhibiting the activation of the Wnt pathway and blocking intestinal tumorigenesis and tumorigenic activity.^20^ In addition, the combination of NCB-0846 and ABT-263 (BCL-X) inhibitor also showed synergistic inhibition of colorectal tumors in the KRAS mutant xenograft model.^36^ The inhibitory effect of NCB-0846 on Wnt target genes also shows good tumor suppressor effects in synovial sarcoma, inducing rapid apoptosis of synovial sarcoma cells.^37^ Similar to colorectal cancer and synovial sarcoma, the Wnt pathway is very important for the occurrence and development of PCa, and KRAS mutations also occur in PCa. The use or combination treatment of NCB-0846 in colorectal cancer and synovial sarcoma can provide a reference for the treatment of PCa. More importantly, NCB-0846 exhibits an inhibitory effect on lung cancer cell metastasis, which is mediated through the epithelial to mesenchymal transition (EMT) mechanism. NCB-0846 blocks the activation of Smad signaling and the induction of EMT by downregulating the expression of transforming growth factor beta (TGF-β) receptor type I.^38^ In PCa, the metastasis and drug resistance caused by EMT have always been problems that trouble people. Interestingly, NCB-0846 can disrupt the transport and secretion of type I procollagen and inhibit the production of matrix proteins to regulate liver fibrosis.^39^ In PCa, matrix proteins can induce tumor cell metastasis and adhesion to bone cells.^39^ Although previous studies have demonstrated that NCB-0846 has a good therapeutic effect in ERG-positive PCa, whether it has the effect of regulating PCa EMT and bone metastasis has not been verified.^13^ Here, our findings demonstrate that AR functions as a transcriptional repressor in TNIK by binding H3K27me3 complex. When AR levels decrease, TNIK phosphorylated EGFR and activating EGFR pathway to escape from castration treatment and promoted CRPC progression. By inhibiting the TNIK/EGFR axis, NCB-0846 showed inhibitory effects on CRPC tumor cells both *in vivo* and *in vitro* and blocked EMT of tumor cells as well as bone metastasis. Through current research, we have reason to believe that NCB-0846 has great potential for the treatment of PCa. These results provide the possibility for the combined treatment of PCa with AR and TNIK, which may be the key to inhibiting the progression of CRPC and provide a reference for the launch of ferroptosis treatment.

### Limitations of the study

In this study, we found that the expression levels of markers of PCa EMT and bone metastasis also changed after inhibiting TNIK using NCB-0846, demonstrating the therapeutic potential of TNIK-targeted therapy in PCa. However, the mechanism by which TNIK regulates the EMT process of PCa and bone metastasis remains unclear, and more phenotypic validation is needed. This is crucial for the development of future PCa therapies. Future research needs to be more specific and targeted to explore the regulation of EMT and bone metastasis by TNIK in PCa.

## STAR★Methods

### Key resources table

**Table:** 

REAGENT or RESOURCE	SOURCE	IDENTIFIER
**Antibodies**		

Anti-TNIK antibody	Cell Signaling Technology	Cat# 32712; RRID: AB_2799027
Anti-TNIK antibody	Bioss	Cat# bs-4168R; RRID: AB_11091303
Anti-p-TNIK antibody	Abcepta	Cat# AP3276a; RRID: AB_637841
Anti-AR antibody	Cell Signaling Technology	Cat# 5153; RRID: AB_10691711
Anti-H3K27me3 antibody	Affinity Biosciences	Cat# DF6941; RRID: AB_2838900
Anti-EZH2 antibody	Affinity Biosciences	Cat# AF5150; RRID: AB_2837636
Anti-EGFR antibody	Cell Signaling Technology	Cat# 4267; RRID: AB_2246311
Anti-p-EGFR antibody	Affinity Biosciences	Cat# AF3048; RRID: AB_2834475
Anti-STAT3 antibody	Affinity Biosciences	Cat# AF6294; RRID: AB_2835144
Anti-p-STAT3 antibody	Cell Signaling Technology	Cat# 4113; RRID: AB_2198588
Anti-ERK antibody	Cell Signaling Technology	Cat# 4695; RRID: AB_390779
Anti-p-ERK antibody	Cell Signaling Technology	Cat# 4370; RRID: AB_2315112)
Anti-NRF2 antibody	Affinity Biosciences	Cat# AF0639; RRID: AB_2833793
Anti-KRAS antibody	Affinity Biosciences	Cat# DF6324; RRID: AB_2838288
Anti-GAPDH antibody	Affinity Biosciences	Cat# AF7021; RRID: AB_2839421
Anti-Myc-tag antibody	Affinity Biosciences	Cat# T0052; RRID: AB_2843446
Anti-Ki67 antibody	Affinity Biosciences	Cat# AF0198; RRID: AB_2834152
Anti-β-catenin antibody	Affinity Biosciences	Cat# AF6266; RRID: AB_2835124
Anti-Vimentin antibody	Affinity Biosciences	Cat# BF8006; RRID: AB_2847777
Anti- E-cadherin antibody	Affinity Biosciences	Cat# BF0219; RRID: AB_2833860
Anti- BMP6 antibody	Affinity Biosciences	Cat# AF5196; RRID: AB_2837682
Anti- BMP7 antibody	Affinity Biosciences	Cat# AF5193; RRID: AB_2837679
Goat polyclonal anti-Rabbit IgG (H+L)	Affinity Biosciences	Cat# S0001; RRID: AB_2839429
Goat polyclonal anti-Mouse IgG (H+L)	Affinity Biosciences	Cat# S0002; RRID: AB_2839430

**Bacterial and virus strains**		

BL-21Lys bacteria	Millipore Sigma	Cat# CMC0015

**Chemicals, peptides, and recombinant proteins**		

MYC-EGFR peptides	Addgene	Cat# 32751
Lipofectamine 2000	ThermoFisher	Cat# 11668019
Dihydrotestosterone	Amersham	N/A
MDV3100	MCE	Cat# HY-70002
Dznep	MCE	Cat# HY-12186
GSK-J1	MCE	Cat# HY-15648
Fetal Bovine Serum	Gibco	Cat# 10099
Crystal violet	LEAGENE	Cat# DZ0053
RIPA lysis buffer	KANGWAY	Cat# 10243

**Critical commercial assays**		

Pierce™ Classic Magnetic IP/Co-IP Kit	ThermoFisher	Cat# 88804
SimpleChIP Plus Enzymatic Chromatin IP Kit	Cell Signaling Technology	Cat# 9005
GeneChip® Eukaryotic Poly-A Control Kit	Affymetrix	Cat# 900433
RiboMinusTM Human Transcriptome Isolation Kit	Invitrogen	Cat# K155001
GeneChip® WT Sense Target Labeling and Control Reagents Kit	Affymetrix	Cat# 902156
GeneChip® WT Terminal Labeling Kit	Affymetrix	Cat# 900671
GeneChip™ Human Gene 1.0 ST Array	ThermoFisher	Cat# 901085
GeneChip® Fluidics Station 450	Affymetrix	Cat# 00-0079
GeneChip® Scanner 3000 7G	Affymetrix	Cat# 00-0210
MTT Kit	Solarbio	Cat# M8180
Lipid Hydroperoxide (LPO) Assay Kit	Jining Shiye	Cat# JN24889

**Experimental models: Cell lines**		

C4-2	ATCC	Cat# CRL-3314
PC3	ATCC	Cat# CRL-1435
22RV1	ATCC	Cat# CRL-2505
LNCaP	ATCC	Cat# CRL-1740

**Experimental models: Organisms/strains**		

BALB/c mice	HFK Bio-Technology	N/A

**Oligonucleotides**		

See Table S3.		

**Recombinant DNA**		

MYC-EGFR-WT	Addgene	Cat# 32751
MYC-EGFR-ECD	Addgene	Cat# 32751
MYC-EGFR-ICD	Addgene	Cat# 32751
TNIK-D171A	This paper	N/A

**Software and algorithms**		

GraphPad Prism 8.2.1 software	GraphPad Prism Software Inc	https://www.graphpad.com/
ImageJ	NIH	https://imagej.nih.gov/ij/
Adobe Illustrator 2023 software	Adobe Systems Inc.	https://www.adobe.com/cn/

### Resource availability

#### Lead contact

Further information and requests for resources and reagents should be directed to and will be fulfilled by the lead contact, Ning Jiang (jiangning@tmu.edu.cn).

#### Materials availability

The plasmids used in this study are available from the lead contact.

#### Data and code availability

• All data reported in this paper will be shared by the lead contact upon request.
• This paper does not report original code.
• Any additional information required to reanalyze the data reported in this paper is available from the lead contact upon request

### Experimental model and study participant details

#### Animal

Four-week-old male BALB/c mice (HFK Bio-Technology Co. Ltd, Beijing) were subcutaneously injected with 2×106 C4-2 cells suspended in 0.1 mL of Matrigel (BD Biosciences) and implanted into the dorsal flank on both sides of the mice. Once the tumors reached a size of approximately 100 mm3, the mice were orally administered either vehicle (10% DMSO in PBS) or NCB-0846 (80 mg/kg of body weight) daily for 10 days by oral gavage (n = 4 mice for each treatment). Tumor volume was measured using digital calipers and estimated using the formula LW2/2, where L represents length of tumor and W represents width. At the end of the study, mice were euthanized, and tumors were extracted and weighed. All procedures involving mice were approved by the University Committee on Use and Care of Animals at Tianjin Medical University and complied with all regulatory standards. The permit number for mouse experiments is SYXK (Jin) 2019-0004.

#### Ethic

CRPC and HSPC tumor tissues as well as paracancerous samples were obtained from patients undergoing radical prostate cancer surgery and examined by certified pathologists. All procedures performed in studies involving human participants were in accordance with the ethical standards of the Research Ethics Committee of The Second Hospital of Tianjin Medical University and with the 1964 Helsinki declaration and its later amendments. ALL written informed consent to participate in the study was obtained from prostate cancer patients for samples to be collected from them (number:KY2021K192).

#### Cell lines and cell culture

The PC cell lines LNCaP, C4-2, 22RV1, and PC3 were obtained from ATCC. The cells were cultured in RPMI 1640 medium supplemented with 10% fetal bovine serum (FBS), 1% penicillin/streptomycin, and 1% glutamine. Dihydrotestosterone (DHT) was acquired from Amersham (Braunschweig, Germany). At the beginning and end of the experiment, the above cell lines were verified through the STR genotyping-based cell line identification service provided by Gene Create Company, and no abnormalities were found. The cells were tested negative for mycoplasma by the mycoplasma detection kit (Gendx, KS201).

### Method details

#### SiRNA

The validated siRNAs were obtained from GenePharma (Shanghai, China). Transfections were conducted using lipo2000 (Thermo Fisher) in accordance with the manufacturer's instructions.

#### Co-immunoprecipitation and western blotting

LNCaP and C4-2 cells were harvested and lysed in lysis buffer containing 150 mM KCl, 75 mM Hepes (pH 7.5), 1.5 mM EGTA, 1.5 mM MgCl2, 10% glycerol, and 0.075% NP-40 supplemented with a protease inhibitor cocktail from Roche (USA). The extracted proteins were precleared using a mixture of protein A–Sepharose (CL-4B; GE Healthcare) and antibody overnight at 4°C. Immunoprecipitates were washed with lysis buffer, resuspended in sample buffer, boiled, and analyzed by SDS-PAGE. Individual samples (40 μg of protein) were separated on an 8% SDS polyacrylamide gel and transferred to PVDF membranes from Millipore (Billerica, MA). The membranes were blocked in a PBS-Tween20 solution with 5% fat-free milk for one hour at room temperature before being incubated overnight at 4°C with appropriate dilutions of specific primary AR or TNIK antibodies. After washing, the blots were incubated with HRP-conjugated anti-rabbit or anti-mouse IgG for one hour. Finally, the blots were developed using an ECL mixture from Vector Laboratories (Burlingame, CA) and visualized by Imager.

#### Chromatin immunoprecipitation

LNCaP cells were cultured in 1640 medium (Invitrogen) with the addition of 10% charcoal-stripped fetal bovine serum (CSF, HyClone, USA) for a duration of 12 hours. DNA cross-linking was performed by adding 1% formaldehyde to the cell cultures at room temperature for a period of 10 minutes, followed by the addition of glycine (final concentration: 0.125 M) for an additional 5 minutes to halt the cross-linking reaction. Cells were lysed using a lysis buffer containing protease inhibitors and sonicated to fragment genomic DNA into sizes ranging from 200 to 1000 base pairs. One-tenth of the cell lysate was used as an input control, while the remaining portion underwent immunoprecipitation using AR or H3K27me3 antibodies. After collecting the immunoprecipitates using protein G-agarose columns, protein-DNA complexes were eluted and heated at a temperature of 65°C to reverse the cross-linking process. Following digestion with proteinase K, DNA fragments were purified using spin columns and analyzed via PCR for a total of 35 cycles under conditions consisting of denaturation at a temperature of94°C for a durationof30 seconds, annealing at55 °C for another thirty seconds and extension at 72°C for one minute each cycle. The specific primer sets targeting a sequence within the human TNIK promoter (as listed in Table S3) were designed accordingly. The resulting PCR products were subsequently electrophoresed ona1.5% agarose gel stained with ethidium bromide and visualized under ultraviolet light.

#### Whole-transcript expression array and microarray image processing

The starting material for generating total RNA/Poly-A RNA controls was 1 μg/μl of total RNA, which was mixed with the GeneChip® Eukaryotic Poly-A Control Kit (Affymetrix, Inc., CA, USA). To enhance sensitivity, the majority of rRNA was eliminated from the total RNA samples prior to target labeling using the RiboMinusTM Human Transcriptome Isolation Kit (Invitrogen, CA, USA). Subsequently, cDNA synthesis was performed according to the manufacturer's instructions using the GeneChip® WT Sense Target Labeling and Control Reagents Kit (Affymetrix, Inc., CA, USA). The resulting sense cDNA was fragmented by UDG (uracil DNA glycosylase) and APE 1 (apurinic/apyrimidinic endonuclease 1), followed by biotin labeling with TdT (terminal deoxynucleotidyl transferase) using a GeneChip® WT Terminal Labeling Kit (Affymetrix, Inc., CA, USA). Once the biotin-labeled sense target DNA was prepared, it underwent hybridization to a gene chip known as The GeneChip® Human Exon 1.0 ST array. Hybridization involved incubating 5 μg of biotinylated target with a GeneChip® Hybridization Wash and Stain Kit and a GeneChip® Fluidics Station 450 (Affymetrix, Inc., CA, USA). Finally, the arrays were scanned using a GeneChip® Scanner 3000 7G(Affymetrix，Inc., CA，USA), and raw data were extracted from scanned images for analysis utilizing Agilent Technologies'GeneSpring GX software version11.5.

#### Immunohistochemistry

The tissue sections were deparaffinized in xylene and rehydrated with graded alcohol. Antigen retrieval was performed under pressure for 5 minutes in citrate buffer (pH adjusted to 6.0). Endogenous peroxidase activity was blocked with 0.3% hydrogen peroxide for 10 minutes, followed by blocking with 1.5% horse serum. Primary antibody incubation was carried out overnight at 4°C in a humidified chamber. After applying Poly-HRP anti-rabbit IgG (30 min), secondary antibody detection was performed using the Ultraview DAB detection kit (Zhongshan Co., China). All immunostained sections were evaluated under a Zeiss microscope (×200). At least ten high-power fields around each malignant gland were assessed and scored.

#### MTT assay

After 48 hours of transfection, a total of 2.0 × 103 cells per well were seeded in 96-well plates and subjected to treatment with either DMSO or erastin at a temperature of 37°C for durations of 24, 48, 72, and 96 hours. Subsequently, each well was supplemented with 30 μl of MTT solution and incubated at a temperature of 37°C for a period specified by the experiment protocol (indicated time). Following this incubation period, the MTT solution was aspirated and replaced with an addition of dimethyl sulfoxide (DMSO) measuring up to a volume of150 μl per well in order to dissolve the formazan crystals. Finally, absorbance measurements were taken at a wavelength of 490 nm using a microplate reader.

#### Determination of lipid peroxidation

After 48 hours of transfection, a total of 5.0 × 106 cells were collected and transferred into a centrifuge tube. Then, 1 ml of extract solution was added to the tube followed by cell lysis and subsequent centrifugation at 4°C for 10 minutes. The resulting supernatant was carefully collected and mixed with the specified reagent (Jining Shiye, JN24889) according to the provided instructions. After thorough mixing, the mixture was heated at 90°C for 40 minutes and then subjected to another round of centrifugation to obtain a final volume of 1 ml supernatant. Finally, the absorbance at 490 nm was measured using a microplate reader.

### Quantification and statistical analysis

The data were presented as mean ± standard deviation. Student's t-test was used to compare two samples, and p values of 0.05 or less were considered statistically significant. Tumor weight was analyzed using GraphPad Prism software. All experiments in our study were repeated at least three times. Statistical significance is indicated with ∗P <.05, ∗∗P < .01, ∗∗∗P < .005.

## Supplemental information

Document S1. Figure S1 and Tables S2 and S3

Table S1. Microarray data for prostate castration tissues and uncastration tissues of mice, related to Figure 1

## References

[1] Siegel R.L., Miller K.D., Jemal A. (2020). Cancer statistics, 2020. CA. Cancer J. Clin..

[2] Azzouni F., Mohler J. (2012). Biology of castration-recurrent prostate cancer. Urol. Clin..

[3] Wang J., Xu L.-F., Liu C., Huang T., Liang C.-Z., Fan Y.-D. (2021). Identifying the role of apolipoprotein A-I in prostate cancer. Asian J. Androl..

[4] Wang Z., Zou J., Zhang L., Liu H., Jiang B., Liang Y., Zhang Y. (2023). Comprehensive analysis of the progression mechanisms of CRPC and its inhibitor discovery based on machine learning algorithms. Front. Genet..

[5] Verras M., Lee J., Xue H., Li T.-H., Wang Y., Sun Z. (2007). The androgen receptor negatively regulates the expression of c-Met: implications for a novel mechanism of prostate cancer progression. Cancer Res..

[6] Liu Y.-N., Liu Y., Lee H.-J., Hsu Y.-H., Chen J.-H. (2008). Activated androgen receptor downregulates E-cadherin gene expression and promotes tumor metastasis. Mol. Cell Biol..

[7] Baniwal S.K., Khalid O., Sir D., Buchanan G., Coetzee G.A., Frenkel B. (2009). Repression of Runx2 by androgen receptor (AR) in osteoblasts and prostate cancer cells: AR binds Runx2 and abrogates its recruitment to DNA. Mol. Endocrinol..

[8] Jiang N., Hjorth-Jensen K., Hekmat O., Iglesias-Gato D., Kruse T., Wang C., Wei W., Ke B., Yan B., Niu Y. (2015). In vivo quantitative phosphoproteomic profiling identifies novel regulators of castration-resistant prostate cancer growth. Oncogene.

[9] Wu N., Wang Y., Wang K., Zhong B., Liao Y., Liang J., Jiang N. (2022). Cathepsin K regulates the tumor growth and metastasis by IL-17/CTSK/EMT axis and mediates M2 macrophage polarization in castration-resistant prostate cancer. Cell Death Dis..

[10] Alaoui-Jamali M.A., Morand G.B., da Silva S.D. (2015). ErbB polymorphisms: insights and implications for response to targeted cancer therapeutics. Front. Genet..

[11] Fu C.A., Shen M., Huang B.C., Lasaga J., Payan D.G., Luo Y. (1999). TNIK, a novel member of the germinal center kinase family that activates the c-Jun N-terminal kinase pathway and regulates the cytoskeleton. J. Biol. Chem..

[12] Salokas K., Liu X., Öhman T., Chowdhury I., Gawriyski L., Keskitalo S., Varjosalo M. (2022). Physical and functional interactome atlas of human receptor tyrosine kinases. EMBO Rep..

[13] Lee R.S., Zhang L., Berger A., Lawrence M.G., Song J., Niranjan B., Davies R.G., Lister N.L., Sandhu S.K., Rubin M.A. (2019). Characterization of the ERG-regulated Kinome in Prostate Cancer Identifies TNIK as a Potential Therapeutic Target. Neoplasia.

[14] Yao Z., Darowski K., St-Denis N., Wong V., Offensperger F., Villedieu A., Amin S., Malty R., Aoki H., Guo H. (2017). A Global Analysis of the Receptor Tyrosine Kinase-Protein Phosphatase Interactome. Mol. Cell.

[15] Chon H.J., Lee Y., Bae K.J., Byun B.J., Kim S.A., Kim J. (2016). Traf2- and Nck-interacting kinase (TNIK) is involved in the anti-cancer mechanism of dovitinib in human multiple myeloma IM-9 cells. Amino Acids.

[16] Zhang Y., Jiang H., Qin M., Su X., Cao Z., Wang J. (2016). TNIK serves as a novel biomarker associated with poor prognosis in patients with pancreatic cancer. Tumour Biol..

[17] Kim J., Moon S.-H., Kim B.T., Chae C.H., Lee J.Y., Kim S.H. (2014). A novel aminothiazole KY-05009 with potential to inhibit Traf2- and Nck-interacting kinase (TNIK) attenuates TGF-β1-mediated epithelial-to-mesenchymal transition in human lung adenocarcinoma A549 cells. PLoS One.

[18] Jin J., Jung H.Y., Wang Y., Xie J., Yeom Y.I., Jang J.-J., Lee K.B. (2014). Nuclear expression of phosphorylated TRAF2- and NCK-interacting kinase in hepatocellular carcinoma is associated with poor prognosis. Pathol. Res. Pract..

[19] Mahmoudi T., Li V.S.W., Ng S.S., Taouatas N., Vries R.G.J., Mohammed S., Heck A.J., Clevers H. (2009). The kinase TNIK is an essential activator of Wnt target genes. EMBO J..

[20] Masuda M., Uno Y., Ohbayashi N., Ohata H., Mimata A., Kukimoto-Niino M., Moriyama H., Kashimoto S., Inoue T., Goto N. (2016). TNIK inhibition abrogates colorectal cancer stemness. Nat. Commun..

[21] Höti N., Lih T.S., Pan J., Zhou Y., Yang G., Deng A., Chen L., Dong M., Yang R.B., Tu C.F. (2020). A Comprehensive Analysis of FUT8 Overexpressing Prostate Cancer Cells Reveals the Role of EGFR in Castration Resistance. Cancers.

[22] Peraldo-Neia C., Cavalloni G., Fenocchio E., Cagnazzo C., Gammaitoni L., Cereda S., Nasti G., Satolli M.A., Aprile G., Reni M. (2018). Prognostic and predictive role of EGFR pathway alterations in biliary cancer patients treated with chemotherapy and anti-EGFR. PLoS One.

[23] Jiang N., Ke B., Hjort-Jensen K., Iglesias-Gato D., Wang Z., Chang P., Zhao Y., Niu X., Wu T., Peng B. (2017). YAP1 regulates prostate cancer stem cell-like characteristics to promote castration resistant growth. Oncotarget.

[24] Chittock E.C., Latwiel S., Miller T.C.R., Müller C.W. (2017). Molecular architecture of polycomb repressive complexes. Biochem. Soc. Trans..

[25] Kim J., Lee Y., Lu X., Song B., Fong K.-W., Cao Q., Licht J.D., Zhao J.C., Yu J. (2018). Polycomb- and Methylation-Independent Roles of EZH2 as a Transcription Activator. Cell Rep..

[26] Masuda M., Yamada T. (2017). The emergence of TNIK as a therapeutic target for colorectal cancer. Expert Opin. Ther. Targets.

[27] Rajput M., Singh R., Singh N., Singh R.P. (2021). EGFR-mediated Rad51 expression potentiates intrinsic resistance in prostate cancer via EMT and DNA repair pathways. Life Sci..

[28] Moehren U., Papaioannou M., Reeb C.A., Hong W., Baniahmad A. (2007). Alien interacts with the human androgen receptor and inhibits prostate cancer cell growth. Mol. Endocrinol..

[29] Liao G., Chen L.Y., Zhang A., Godavarthy A., Xia F., Ghosh J.C., Li H., Chen J.D. (2003). Regulation of androgen receptor activity by the nuclear receptor corepressor SMRT. J. Biol. Chem..

[30] Berrevoets C.A., Umar A., Trapman J., Brinkmann A.O. (2004). Differential modulation of androgen receptor transcriptional activity by the nuclear receptor co-repressor (N-CoR). Biochem. J..

[31] D'Abronzo L.S., Bose S., Crapuchettes M.E., Beggs R.E., Vinall R.L., Tepper C.G., Siddiqui S., Mudryj M., Melgoza F.U., Durbin-Johnson B.P. (2017). The androgen receptor is a negative regulator of eIF4E phosphorylation at S209: implications for the use of mTOR inhibitors in advanced prostate cancer. Oncogene.

[32] Cai C., He H.H., Chen S., Coleman I., Wang H., Fang Z., Chen S., Nelson P.S., Liu X.S., Brown M., Balk S.P. (2011). Androgen receptor gene expression in prostate cancer is directly suppressed by the androgen receptor through recruitment of lysine-specific demethylase 1. Cancer Cell.

[33] Traish A.M., Morgentaler A. (2009). Epidermal growth factor receptor expression escapes androgen regulation in prostate cancer: a potential molecular switch for tumour growth. Br. J. Cancer.

[34] Pham T.C.P., Dollet L., Ali M.S., Raun S.H., Møller L.L.V., Jafari A., Ditzel N., Andersen N.R., Fritzen A.M., Gerhart-Hines Z. (2023). TNIK is a conserved regulator of glucose and lipid metabolism in obesity. Sci. Adv..

[35] Liang J., Liao Y., Wang P., Yang K., Wang Y., Wang K., Zhong B., Zhou D., Cao Q., Li J. (2023). Ferroptosis landscape in prostate cancer from molecular and metabolic perspective. Cell Death Dis..

[36] Jung H.R., Oh Y., Na D., Min S., Kang J., Jang D., Shin S., Kim J., Lee S.E., Jeong E.M. (2021). CRISPR screens identify a novel combination treatment targeting BCL-XL and WNT signaling for KRAS/BRAF-mutated colorectal cancers. Oncogene.

[37] Sekita T., Yamada T., Kobayashi E., Yoshida A., Hirozane T., Kawai A., Uno Y., Moriyama H., Sawa M., Nagakawa Y. (2020). Feasibility of Targeting Traf2-and-Nck-Interacting Kinase in Synovial Sarcoma. Cancers.

[38] Sugano T., Masuda M., Takeshita F., Motoi N., Hirozane T., Goto N., Kashimoto S., Uno Y., Moriyama H., Sawa M. (2021). Pharmacological blockage of transforming growth factor-β signalling by a Traf2- and Nck-interacting kinase inhibitor, NCB-0846. Br. J. Cancer.

[39] Buchl S.C., Hanquier Z., Haak A.J., Thomason Y.M., Huebert R.C., Shah V.H., Maiers J.L. (2022). Traf2 and NCK Interacting Kinase Is a Critical Regulator of Procollagen I Trafficking and Hepatic Fibrogenesis in Mice. Hepatol. Commun..

