# Supplementary material for: TNIK drives castration-resistant prostate cancer via phosphorylating EGFR
Source: iScience. 2023 Dec 12;27(1):108713. doi: 10.1016/j.isci.2023.108713 (PMC10788198; doi:10.1016/j.isci.2023.108713)
Supplement: Document S1. Figure S1 and Tables S2 and S3 [file mmc1.pdf]

iScience, Volume *27*

Supplemental information

TNIK drives castration-resistant prostate cancer via phosphorylating EGFR

Jianing Guo, Jiaming Liang, Youzhi Wang, Tao Guo, Yihao Liao, Boqiang Zhong, Shuyue Guo, Qian Cao, Junbo Li, Amilcar Flores-Morales, Yuanjie Niu, and Ning Jiang

Supplemental materials

Supplemental figures

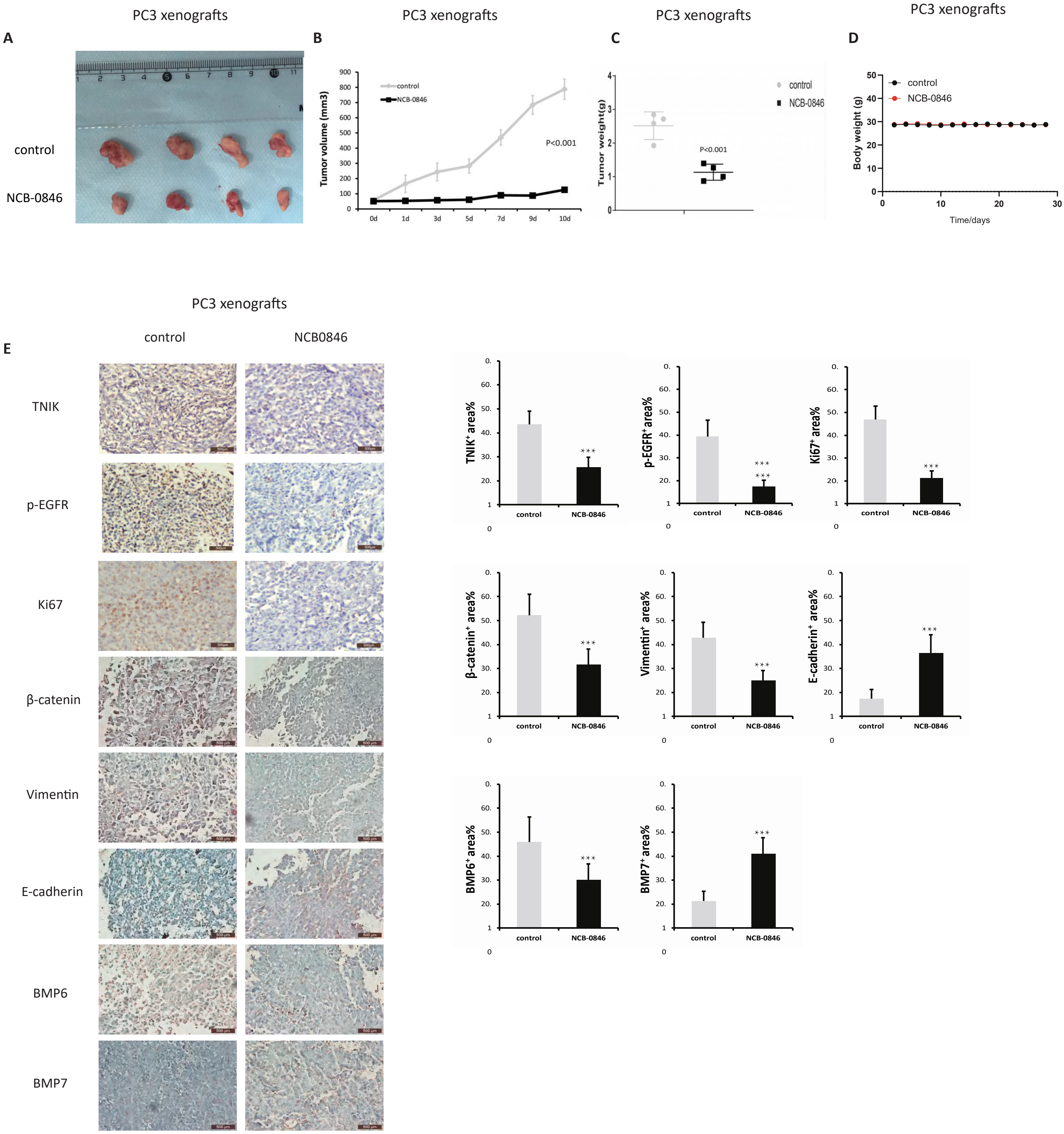

Supplemental Figure 1: Targeting TNIK suppresses CRPC tumor progression in vivo, related to Figure 6. (A) PC3 cells were implanted subcutaneously in male BALB/c mice. When tumors became palpable, mice administered daily by oral gavage either with vehicle (10% DMSO in PBS) or NCB0846 (80 mg/kg of body weight) for 10 days (n = 4 mice for each treatment). Tumor volumes were measured with calipers. (B) Tumor size of xenografts of the above represented the growth of tumor over 10 days (n = 4) in athymic nude mice ($p < 0.001$). Data are shown as mean ± SD. (C) tumor weight of the control mice tumors and NCB-0846-treated mice tumors ($p < 0.001$). Data are shown as mean ± SD. (D) Body weight of nude mice after implantation of control or PC3 xenografts and treatment with vehicle or NCB-0846 for 4 weeks. (E) Quantitation of Ki-67, TNIK, p-EGFR, β-catenin, Vimentin, E-cadherin, BMP6 and BMP7 expressions in PC3 xenograft tumors from each group, specimens were got at 10 days post treatment. Scale bars: 500 μm. The IHC was scored according to number of cells expressing the indicated proteins and statistical analysis was performed (non-parametric Kruskal-Wallis test) in order to determine significance. Data are shown as mean ± SD. ***$p < 0.005$.

Supplemental tables

Table S2 The profile of genes differentially expressed in LNCaP-CR vs LNCaP-HS, related to Figure 4

Description	GeneRatio	BgRatio	pvalue	p.adjust	qvalue	geneID	Count
MAPK signaling pathway	49/828	294/8035	0.000424771	0.003465234	0.002459199	369/408/673/994/1398/139	49
Human cytomegalovirus infection	43/828	225/8035	4.13E-05	0.00058227	0.000413224	109/23365/578/581/595/13	43
Regulation of actin cytoskeleton	42/828	213/8035	2.35E-05	0.000454348	0.000322441	10152/60/87/324/369/2909	42
Autophagy - animal	41/828	137/8035	1.25E-10	3.88E-08	2.76E-08	84335/55626/55102/598/10	41
Human immunodeficiency virus 1 infection	36/828	212/8035	0.001703074	0.010774547	0.007646453	8907/8905/9582/578/581/5	36
RNA transport	35/828	180/8035	0.000148189	0.001640668	0.001164345	1965/8661/8662/8665/8669	35
mTOR signaling pathway	34/828	155/8035	1.41E-05	0.000335597	0.000238165	84335/673/1857/1975/5760	34
Wnt signaling pathway	33/828	160/8035	6.97E-05	0.00079976	0.000567571	324/817/818/595/57680/13	33
Cell cycle	31/828	124/8035	1.94E-06	8.58E-05	6.09E-05	25/9184/595/8555/994/101	31
Hippo signaling pathway	31/828	157/8035	0.00026252	0.002625197	0.001863043	60/324/332/657/659/595/1	31
Cellular senescence	31/828	160/8035	0.000371942	0.003465234	0.002459199	823/595/1017/1870/2305/2	31
FoxO signaling pathway	30/828	131/8035	1.86E-05	0.000411686	0.000292164	369/673/595/1017/1387/20	30
AMPK signaling pathway	27/828	120/8035	6.68E-05	0.000796311	0.000565124	32/79602/84335/10645/595	27
Oxytocin signaling pathway	27/828	154/8035	0.003919505	0.020711251	0.014698307	60/109/817/818/10645/595	27
ErbB signaling pathway	26/828	85/8035	1.96E-07	1.22E-05	8.63E-06	25/27/369/673/817/818/86	26
EGFR tyrosine kinase inhibitor resistance	22/828	79/8035	9.44E-06	0.000266073	0.000188826	369/558/581/598/673/2247	22
TGF-beta signaling pathway	20/828	94/8035	0.001206071	0.007954938	0.00564544	90/91/657/659/1387/2033/	20
Prostate cancer	20/828	97/8035	0.001806115	0.011197911	0.007946904	369/673/595/1017/1387/18	20
Renal cell carcinoma	19/828	69/8035	4.51E-05	0.00060803	0.000431505	369/673/1387/1398/1399/2	19
p53 signaling pathway	18/828	72/8035	0.00027267	0.002641488	0.001874604	84883/581/598/595/1017/9	18
Longevity regulating pathway	18/828	89/8035	0.003728886	0.020279908	0.014392193	109/79602/84335/581/1064	18
mRNA surveillance pathway	18/828	91/8035	0.004774558	0.024264147	0.017219717	51692/11052/79869/26528/	18
PD-L1/PD-1 checkpoint in cancer	17/828	89/8035	0.00859095	0.038597021	0.027391434	1459/27436/3845/5605/421	17
Mitophagy - animal	14/828	65/8035	0.005591466	0.027513561	0.019525753	55626/598/1459/2309/3845	14
Prolactin signaling pathway	14/828	70/8035	0.010934901	0.047743934	0.033882792	595/2309/2885/2932/3845/	14
DNA replication	13/828	36/8035	3.25E-05	0.000559901	0.000397349	2237/3978/4171/4172/4173	13
VEGF signaling pathway	13/828	59/8035	0.00610021	0.029547893	0.020969473	3845/5605/5594/7867/4773	13
Bladder cancer	10/828	41/8035	0.007372722	0.034529342	0.024504694	369/673/595/1870/3845/56	10

Table S3 Oligonucleotides used in this study, related to STAR Methods

		Forword primer	Reverse primer
**q-PCR**			
	TNIK	5'-ACAGTGGCTGTCAGCGACATAC-3'	5'-ATACTGCCGCTGAAACTGTCCG-3'
	GAPDH	5'-CCAGCAAGAGCACAAGAGGAAGAG-3'	5'-CAAGGGGTCTACATGGCAACTGTG-3'
	PTCH1	5'-GCTGCACTACTTCAGAGACTGG-3'	5'-CACCAGGAGTTTGTAGGCAAGG-3'
	RFWD3	5'-ATCCGTGGACTGGCGTTTAGCA-3'	5'-GCCTCATCAAGACACCAGCAAC-3'
	CTNNB1	5'-CACAAGCAGAGTGCTGAAGGTG-3'	5'-GATTCCTGAGAGTCCAAAGACAG-3'
	FLNA	5'-CAACAAGTTCACTGTGGAGACCA-3'	5'-TGTAGGTGCCAGCCTCATAAGG-3'
	NUCKS1	5'-GACGATAGTGACTATGGCAGTTC-4'	5'-CCTTTCACTGGACTTGGCGTCA-4'
**ChIP-PCR**			
	TNIK	5'-CAACTCGGTTGTTTCCGTG-3'	5'-TGGGAAGGTTTGCCAGAAG-3'
